# Vitamin D, systemic inflammation, and motoric cognitive risk: exploring the risk factors and mediation pathways

**DOI:** 10.3389/fragi.2026.1748389

**Published:** 2026-04-28

**Authors:** Yunhe Xu, Pei Qin, Ningqi Kang, Ying Li, Lijiao Wang

**Affiliations:** 1 Second Clinical Medical College, Chengde Medical University, Chengde, Hebei, China; 2 Department of Geriatrics, Chengde Central Hospital, Chengde, Hebei, China

**Keywords:** 25-hydroxy vitamin D, mediation analysis, motoric cognitive risk syndrome, systemic immune-inflammation index, systemic inflammation

## Abstract

**Introduction:**

Motoric cognitive risk (MCR) syndrome, defined by subjective cognitive complaints and slow gait, is a predementia condition associated with multiple adverse outcomes. Although vitamin D deficiency and systemic inflammation have been implicated in cognitive and motor decline, their interaction in the context of MCR remains unclear.

**Methods:**

This cross-sectional study included 312 hospitalized adults aged ≥60 years. Serum 25(OH)D levels, systemic immune-inflammation index (SII), and standardized clinical assessments were obtained within 48 h of admission. Multivariable regression and mediation analyses were performed to evaluate associations and underlying pathways.

**Results:**

The prevalence of MCR was 17.9%. Older age, greater comorbidity burden, poor sleep quality, lower 25(OH)D levels, and higher SII were independently associated with MCR. Mediation analysis showed that systemic inflammation accounted for approximately one-quarter of the association between lower 25(OH)D and higher MCR risk, with the strongest indirect effect observed at 25(OH)D ≤ 20 ng/mL.

**Discussion:**

Vitamin D deficiency and systemic inflammation are important determinants of MCR. The identified vitamin D–inflammation pathway may contribute to motoric cognitive vulnerability and provide insights for early risk stratification and preventive strategies in geriatric populations.

## 1 Introduction

Cognitive decline and gait disturbances have become major public health concerns with the accelerating population aging. Motoric cognitive risk (MCR), which is defined by subjective cognitive complaints and slow gait in the absence of dementia, is a simple and clinically accessible predementia marker linked to increased risks of dementia, falls, functional decline, and mortality (Verghese et al., 2013; Shen et al., 2020; Mullin et al., 2021; Huang et al., 2024). Despite its clinical significance, the biological pathways underlying MCR remain insufficiently clarified. Emerging evidence suggests that MCR reflects concurrent impairments in cognitive and motor control systems, with chronic low-grade inflammation considered a potential contributing factor (Groeger et al., 2022). In this context, elevated inflammatory markers have been associated with poorer cognitive performance and a higher risk of cognitive impairment (Serna et al., 2025; Custodero et al., 2022). Furthermore, inflammation has been linked to vascular and structural changes in the brain, including neurovascular dysfunction and white-matter injury (Rajeev et al., 2022), which are considered important substrates for both cognitive impairment and gait slowing. Previous studies have also reported that inflammatory activity may be accompanied by vascular alterations, such as increased carotid intima-media thickness, which, in turn, are associated with cognitive impairment (İmre et al., 2023a). These findings indicate that chronic low-grade inflammation may contribute to MCR through shared neural and vascular mechanisms. The systemic immune-inflammation index (SII), which is calculated from neutrophil, lymphocyte, and platelet counts, provides a stable indicator of the inflammatory burden and has demonstrated prognostic value across diverse conditions (Merchant et al., 2023; Algul and Kaplan, 2024). Compared with single inflammatory markers, SII may better reflect the overall inflammatory status; however, its association with MCR remains insufficiently characterized. Vitamin D, assessed using circulating 25(OH)D, also influences cognitive and motor function through neuroprotective and anti-inflammatory mechanisms (Algul and Kaplan, 2024; Cashman et al., 2017; Gu et al., 2023). It has also been implicated in neuroimmune regulation and broader brain function, including the modulation of inflammatory processes and neural signaling (Wassif et al., 2023), with lower vitamin D levels reported to be associated with neuropsychiatric symptoms and mood dysregulation (İmre et al., 2023b). However, findings on its association with MCR remain inconsistent, and the potential mediating role of systemic inflammation has not been fully elucidated (Santangelo et al., 2021; Conti and Kempuraj, 2016). Using data from older inpatients, in this study, we examined the correlates of MCR and evaluated the joint roles of SII and 25(OH)D within a unified framework. A mediation model was applied to test whether systemic inflammation mediates the link between the vitamin D status and MCR.

## 2 Materials and methods

### 2.1 Study population and selection criteria

This study included 364 consecutive older inpatients admitted to the Department of Internal Medicine at Chengde Central Hospital between 1 January and 30 June 2025. The inclusion criteria were age ≥60 years; ability to complete the subjective cognitive complaint assessment; ability to safely perform a 4-m usual-pace gait test with customary assistive devices if required; and completion of serum 25(OH)D testing, complete blood counts for SII, and other standardized assessments within 48 h of admission, with all the core variables available or eligible for multiple imputation.

The exclusion criteria were a documented diagnosis of dementia or cognitive impairment severe enough to preclude interview; acute delirium or encephalopathy at assessment; recent neurological or musculoskeletal conditions likely to interfere with MCR classification or gait, such as stroke or TIA within 3 months, advanced Parkinson’s disease, normal-pressure hydrocephalus, or lower-limb fracture or major surgery within 3 months; medical instability contraindicating the gait test, including hypoxemia or active chest pain; and conditions or treatments that could substantially affect CBC-derived indices, including active systemic infection requiring antibiotics, hematologic malignancy, recent cytotoxic chemotherapy, systemic glucocorticoids, granulocyte colony-stimulating factor, or recent transfusion.

### 2.2 Collection of impact factors

Data for this study were obtained from the electronic medical record and a structured interviewer-administered questionnaire during the index admission. Information collected included demographics (age, sex, height, weight, residence, living arrangement, and weekly physical activity), comorbidities and current medications verified against medical records, and routine laboratory tests. Fasting venous blood was drawn within 48 h of admission to measure serum 25(OH)D, complete blood count parameters, and lipid profiles, all of which were processed in the hospital’s central laboratory according to standardized procedures. MCR was defined as the coexistence of a subjective cognitive complaint and slow gait without dementia (Verghese et al., 2013). Gait speed was assessed using a standardized 4-m walk, and values below 1.0 m/s were classified as slow.

Data management followed pre-specified procedures to ensure consistency, including uniform cleaning, formatting, and variable derivation. BMI was calculated as the weight divided by height squared, and the comorbidity burden was quantified using the age-adjusted Charlson Comorbidity Index (Klabunde et al., 2000). Sleep quality was assessed using the Pittsburgh Sleep Quality Index, with scores ≥7 indicating poor sleep (Leng et al., 2020). The SII was calculated from platelet, neutrophil, and lymphocyte counts and then z-standardized for analysis (Feng et al., 2017). Educational level was classified as ≤6 or >6 years (Qin et al., 2022; Lam et al., 2008); polypharmacy was defined as the concurrent use of five or more prescription medications (Levy, 2017); and insufficient physical activity was defined as <150 min per week of moderate-intensity activity according to the WHO guidelines (Bull et al., 2004). Cerebral small vessel disease (CSVD) burden was scored on a scale of 0–4 based on standard imaging criteria and categorized as mild or moderate-to-severe for analysis (Staals et al., 2014). Quality control procedures included verification against source documents and automated logic and range checks, with additional details regarding missing data handling provided in the Statistical Analysis section.

### 2.3 Study design and workflow

**FIGURE 1:**
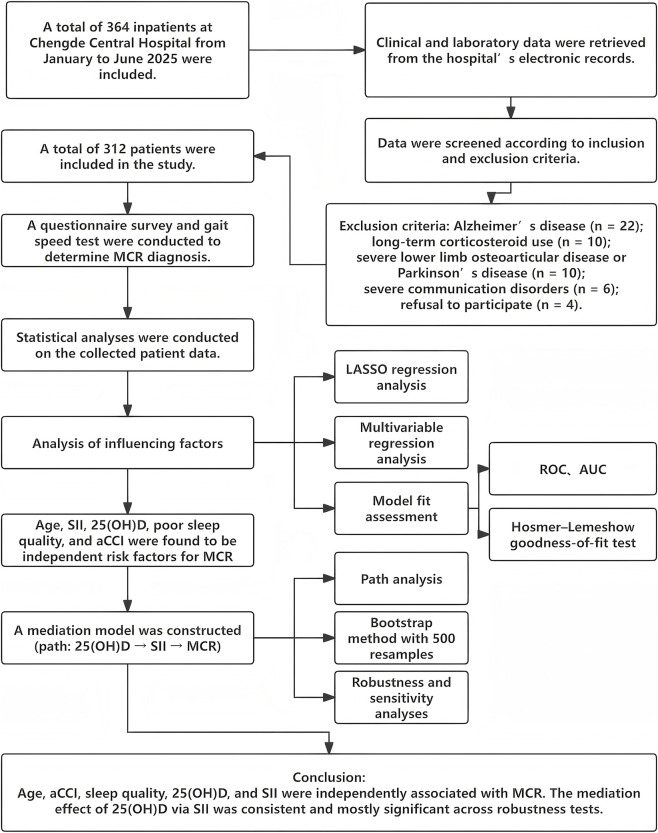
Analytical workflow for variable selection, regression modeling, and mediation analysis.

This study followed a pre-specified analytical workflow. After data extraction and cleaning, continuous variables were z-standardized, and the SII was derived from platelet, neutrophil, and lymphocyte counts. The analysis pipeline consisted of four major steps: variable screening using LASSO logistic regression with 10-fold cross-validation (Verghese et al., 2013); multivariable logistic regression to identify independent correlates of MCR (Shen et al., 2020); model performance evaluation, including discrimination, calibration, internal validation, and decision-curve analysis (Mullin et al., 2021); and mediation analysis under the counterfactual framework to estimate total, direct, and indirect effects of serum 25(OH)D on MCR through SII (Huang et al., 2024). Sensitivity analyses and stratified mediation analyses were conducted to assess robustness. The full workflow is illustrated in Figure 1.

### 2.4 Sample size

This was an exploratory, cross-sectional study based on consecutively enrolled inpatients. The sample size was determined by the number of eligible participants during the study period; therefore, a formal sample size calculation was not performed. Given the number of events and variables included in the regression models, the sample size was deemed adequate for the analyses conducted.

### 2.5 Statistical analysis

All analyses were performed in R version 4.3.2. The dataset was first checked for missing values; variables with minimal missingness were imputed using multiple imputation, while variables with substantial missingness were excluded to reduce bias. Analyses were conducted on the imputed datasets, and pooled estimates were obtained using Rubin’s rules. Normally distributed variables were summarized as the mean ± SD and compared using independent-samples t tests; non-normally distributed variables were summarized as the median (IQR) and compared using the Mann–Whitney U test, including sex-based comparisons of serum 25(OH)D and SII_z. Categorical variables were compared using chi-square or Fisher’s exact tests. A two-sided *p* < 0.05 was considered statistically significant.

LASSO logistic regression was applied to screen candidate predictors after z-standardizing continuous variables, with the penalty parameter selected by 10-fold cross-validation using the one-standard-error rule. Variables with non-zero coefficients were entered into multivariable logistic regression to estimate the adjusted odds ratios with 95% confidence intervals (CIs). Model diagnostics included assessment of logit-linearity using restricted cubic splines, multicollinearity using variance inflation factors, and influential observations using Cook’s distance. The model performance was evaluated using ROC curves and AUC with DeLong CIs, and calibration was examined using the Hosmer–Lemeshow test, calibration intercept and slope, and a loess calibration curve. Weighted models using inverse-prevalence weights were additionally fitted to assess robustness to the outcome imbalance. Internal validation with 1,000 bootstrap resamples yielded optimism-corrected performance estimates, and decision curve analysis was used to evaluate clinical utility.

Mediation analysis evaluated whether serum 25(OH)D influenced MCR indirectly through the SII_z. A linear regression model was fitted for the mediator, and a logistic regression model was used for the outcome, with both adjusted for age, aCCI, sleep quality, and variables retained during screening. Parametric g-computation was used to estimate the total, direct, and indirect effects and the proportion mediated. Because multiple imputation was applied, the mediation models were run within each imputed dataset and pooled using Rubin’s rules, with 95% confidence intervals obtained from 1,000 bootstrap samples. Sensitivity analyses were conducted by replacing the logistic link with a probit link, using the 25th versus 75th percentile contrast of 25(OH)D, adding a quadratic term to assess non-linearity, and trimming the extreme 1% of values for 25(OH)D and SII_z. Each analysis incorporated 1,000 bootstrap resamples. To assess potential heterogeneity, stratified mediation analyses were performed using a 25(OH)D cutoff of 20 ng/mL and repeating the full mediation workflow within each stratum.

## 3 Results

Among the 312 patients included in the analytic cohort, the prevalence of MCR was 17.9%. The participants were older adults with a balanced distribution of sex and residential background. The demographic and lifestyle characteristics, including sleep quality, physical activity, education level, and living arrangement, varied across individuals. Clinical indicators such as BMI, comorbidity burden, serum 25(OH)D levels, lipid profiles, and the standardized SII also showed wide ranges. Imaging-based CSVD burden and medication use, including polypharmacy, demonstrated similar variability. Sex-based univariate comparisons showed no significant differences between male and female individuals in serum 25(OH)D levels or SII_z (all *p* > 0.05). These baseline characteristics are summarized in Table 1 and provide the descriptive context for the subsequent analyses.

**TABLE 1:** Patient demographics and baseline characteristics.

Characteristic		N = 312
MCR	No	256 (82.1%)
	Yes	56 (17.9%)
Age	​	73 (67, 78)
BMI	​	25.3 (22.8, 28.3)
aCCI	​	6.0 (4.0, 9.0)
SII_z	​	−0.33 (−0.75, 0.45)
25(OH)D (ug/mL)	​	20 (15, 24)
ΔGrip strength	​	−1.85 (−3.50, −0.40)
TC (mmol/L)	​	4.40 (3.70, 5.30)
Sex	Female	187 (59.9%)
	Male	125 (40.1%)
Residence	Rural	96 (30.8%)
	Urban	216 (69.2%)
CSVD score	<2	180 (57.7%)
	≥2	132 (42.3%)
Living arrangement	Family	249 (79.8%)
	Living alone	63 (20.2%)
Sleep quality	Poor	178 (57.1%)
	Good	134 (42.9%)
Years of education	≤6 years	199 (63.8%)
	>6 years	113 (36.2%)
Physical activity	<150 min/week	159 (51.0%)
	≥150 min/week	153 (49.0%)
Polypharmacy	≥5	138 (44.2%)
	<5	174 (55.8%)

n (%)/Median (Q1, Q3).

Difference between the left and right handgrip strength.

### 3.1 Screening of potential impact factors

**FIGURE 2:**
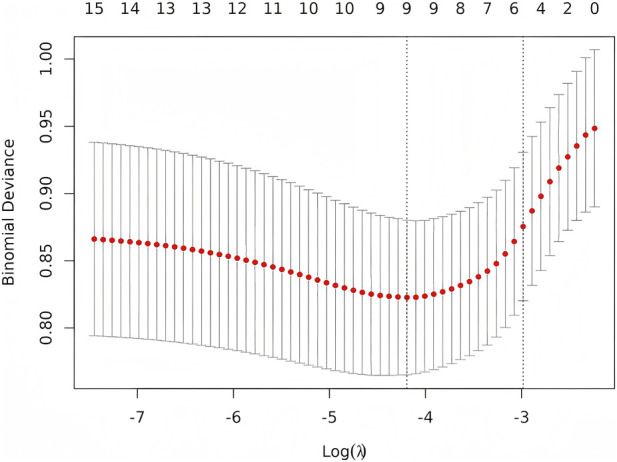
Ten-fold cross-validated error versus log(λ) for the LASSO model.

**FIGURE 3:**
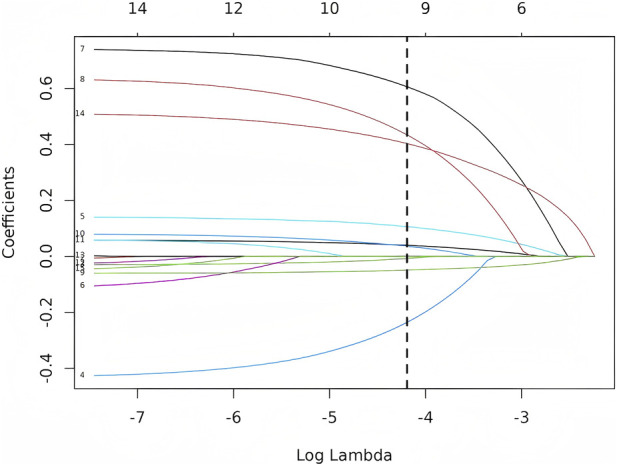
LASSO coefficient paths for all candidate predictors across log(λ).

Using MCR status as the outcome, all clinically relevant variables were first entered into LASSO logistic regression with 10-fold cross-validation. The penalty parameter selected by the 1-SE rule identified a reduced subset of predictors, which were subsequently included in the multivariable logistic regression model for adjusted effect estimation. The variables retained by LASSO and carried forward into the final model are presented in Figures 2, 3.

### 3.2 Multivariable analysis

Multivariable logistic regression showed that older age, higher aCCI, poor sleep quality, and elevated SII_z were independently associated with increased odds of MCR, while higher serum 25(OH)D was associated with lower odds. BMI, handgrip strength deficit, CSVD category, and years of education were not significant predictors. The full results are provided in Table 2.

**TABLE 2:** Multifactor regression results.

Characteristic		OR	95% CI	*p*-value
Age	​	1.06	1.01, 1.11	0.016
BMI	​	0.97	0.89, 1.05	0.451
CSVD score	<2	—	—	​
	≥2	0.65	0.32, 1.32	0.236
aCCI	​	1.15	1.04, 1.28	0.006
Sleep quality	Good	—	—	​
	Poor	2.12	1.04, 4.29	0.037
Years of education	>6 years	—	—	​
	≤6 years	1.90	0.98, 3.69	0.058
25(OH)D	​	0.94	0.89, 0.99	0.034
ΔGrip strength	​	1.08	0.96, 1.21	0.181
SII_z	​	1.65	1.22, 2.24	0.001

Abbreviations: CI, confidence interval; OR, odds ratio.

### 3.3 Model calibration and discrimination

The model showed concordance between the predicted and observed MCR probabilities, and the Hosmer–Lemeshow test indicated no evidence of lack of fit. The calibration results are summarized in Table 3.

**TABLE 3:** Hosmer–Lemeshow goodness-of-fit test.

χ^2^	df	*p*
7.560	8	0.478

**FIGURE 4:**
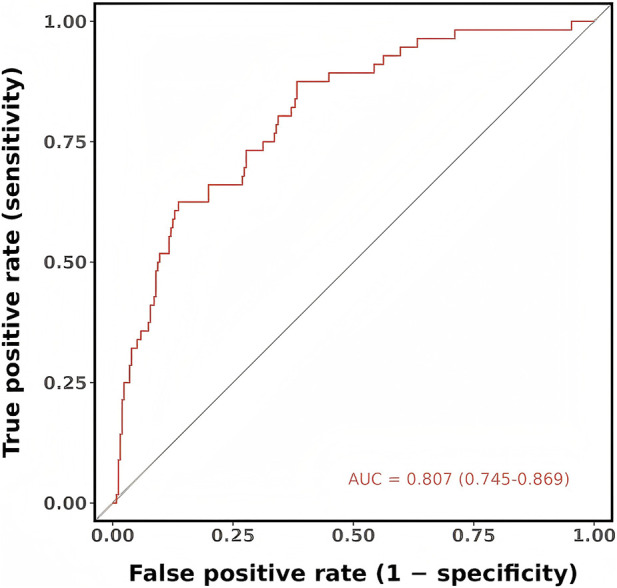
ROC curve for the multivariable logistic regression model predicting MCR.

The multivariable model showed good discrimination, with an AUC value of 0.807 for distinguishing MCR cases from non-cases, as presented in Figure 4.

### 3.4 Analysis of the mediating effect of 25(OH)D on MCR through inflammatory pathways

Based on the variables independently associated with MCR, we examined whether the relationship between serum 25(OH)D and MCR operated partly through systemic inflammation. Systemic inflammation was indexed by the SII and implemented as a z-standardized variable. Guided by the LASSO-selected predictors and multivariable regression results, a mediation model was specified with 25(OH)D as the exposure, SII_z as the mediator, and MCR as the outcome while adjusting for age, aCCI, and sleep quality.

#### 3.4.1 Primary analysis

After covariate selection, the mediation model was adjusted for age, aCCI, and sleep quality. Serum 25(OH)D levels ranged widely (10th–90th percentile: 11.60 ng/mL–27.75 ng/mL). Participants with lower 25(OH)D had a higher prevalence of MCR (*p* < 0.01). In the mediation model, lower 25(OH)D was associated with higher SII_z (β = −0.026, 95% CI: −0.042 to −0.010), and higher SII_z was associated with increased odds of MCR (β = 0.65, 95% CI: 0.30 to 1.00), independent of 25(OH)D. The direct association between 25(OH)D and MCR also remained significant (β = −0.122, 95% CI: −0.238 to −0.005).

**FIGURE 5:**
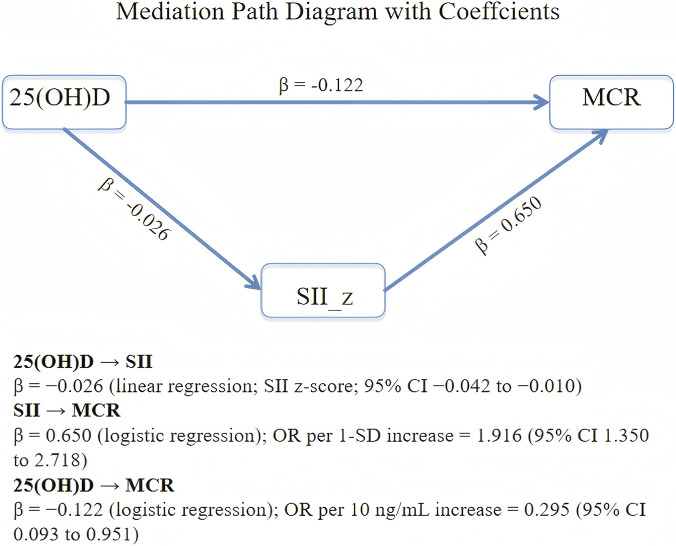
Analysis of the mediating effect of 25(OH)D on MCR via SII_z.

**FIGURE 6:**
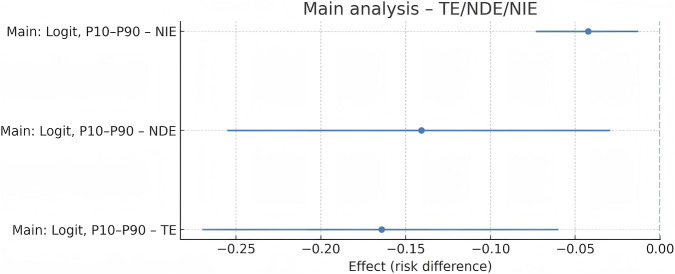
Forest plot of the mediation effects (TE, NDE, and NIE) on the risk-difference scale.

Using parametric g-computation with 1,000 bootstrap samples, the total effect of 25(OH)D on MCR was −0.165 (95% CI: −0.282 to −0.048), the natural direct effect was −0.122 (95% CI: −0.238 to −0.005), and the natural indirect effect via SII_z was −0.043 (95% CI: −0.080 to −0.014). The proportion mediated was 26.0% (95% CI: 9.9% to 43.2%), with the indirect effect confidence interval excluding 0, indicating significant partial mediation. The mediation pathway diagram and effect estimates are shown in Figures 5, 6.

#### 3.4.2 Sensitivity analyses

**FIGURE 7:**
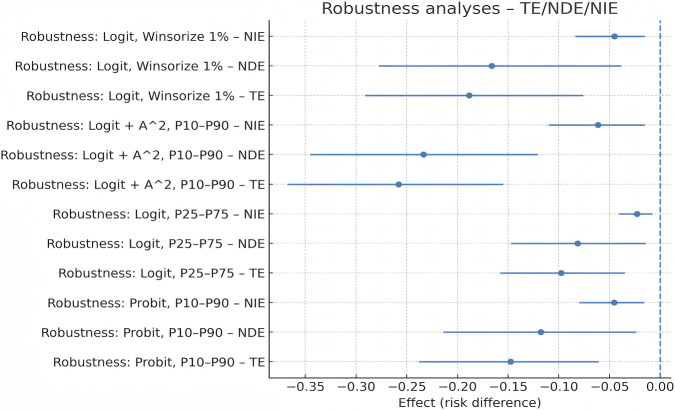
Robustness analyses: forest plot of the mediation effects (TE, NDE, and NIE).

With age, aCCI, and sleep quality held constant, four sensitivity specifications were conducted, with the effects estimated on the risk-difference scale and 95% CIs obtained from 1,000 bootstrap resamples. Using a probit link, the TE was −0.158 (95% CI: −0.278 to −0.042), the NDE was −0.116 (−0.233 to −0.003), the NIE was −0.042 (−0.079 to −0.013), and the PM was 26.6% (10.1%–44.0%). When contrasting the 25th versus 75th percentile of 25(OH)D (15.80 vs. 24.30 ng/mL), the TE was −0.112 (−0.206 to −0.018), the NDE was −0.082 (−0.176 to −0.004), the NIE was −0.030 (−0.059 to −0.009), and the PM was 26.8% (9.8%–46.1%). Incorporating a quadratic term for 25(OH)D produced a TE of −0.161 (−0.280 to −0.044), an NDE of −0.118 (−0.235 to −0.005), an NIE of −0.043 (−0.080 to −0.015), and a PM of 26.7% (10.0%–44.4%). Trimming the extreme 1% of 25(OH)D and SII_z yielded similar results (TE −0.156, NDE −0.114, NIE −0.042, and PM 26.5%). Across all specifications, the NIE remained negative, with confidence intervals excluding 0, and the PM consistently clustered at approximately 26%–27%. These findings indicate that the mediation effect was robust across variations in link function, exposure contrast, nonlinearity, and outlier handling, as shown in Figure 7.

#### 3.4.3 Stratified analysis

To examine heterogeneity based on vitamin D status, participants were stratified at 25(OH)D = 20 ng/mL (≤20 vs. > 20 ng/mL), and mediation analysis was repeated within each subgroup using the same covariate set and estimation approach. Among individuals with low 25(OH)D (≤20 ng/mL), the TE was −0.395 (95% CI: −0.587 to −0.203), the NDE was −0.289 (−0.496 to −0.082), the NIE was −0.106 (−0.194 to −0.027), and the PM was 26.8% (10.4%–45.6%), with the indirect effect confidence interval excluding 0.

**FIGURE 8:**
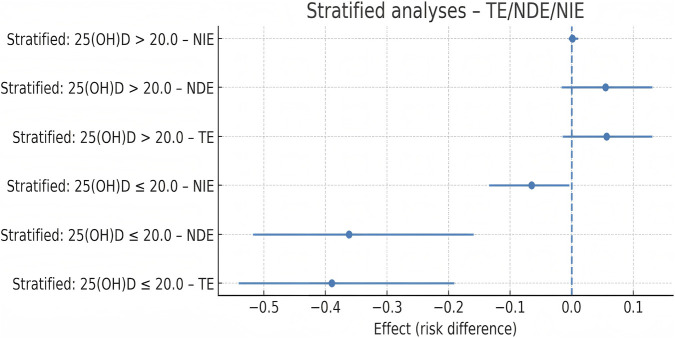
Stratified mediation analysis by the vitamin D status.

In participants with higher 25(OH)D (>20 ng/mL), the TE was −0.092 (95% CI: −0.210 to 0.026), the NDE was −0.070 (−0.188 to 0.049), the NIE was −0.022 (−0.062 to 0.010), and the PM was 23.9% (−3.8% to 51.7%), with the indirect effect confidence interval crossing 0. The mediation effect was, therefore, present in the low-vitamin D subgroup but not in the higher-vitamin D subgroup, as shown in Figure 8.

## 4 Discussion

As population aging accelerates, identifying individuals at increased risk before the development of dementia has become an important public health objective. MCR, defined by subjective cognitive complaints and slow gait in the absence of dementia, is associated with multiple adverse outcomes, including dementia, falls, functional decline, hospitalization, and mortality (Huang et al., 2024), and it has been proposed as a practical early-screening phenotype. In this inpatient cohort (n = 312), LASSO followed by multivariable logistic regression identified older age, higher comorbidity burden, poor sleep quality, lower serum 25(OH)D, and higher SII_z as independent correlates of MCR. These findings emphasize the multifactorial nature of MCR and provide a basis for risk stratification and targeted multi-domain prevention strategies in older adults.

### 4.1 Common determinants of MCR

Age emerged as one of the strongest correlates of MCR, with a 6% increase in odds per additional year (OR = 1.06, 95% CI: 1.01 to 1.11, and *p* < 0.05). This finding is consistent with neuroimaging evidence showing age-related atrophy and white-matter disruption in regions supporting motor and cognitive control (Sathyan et al., 2024; Blumen et al., 2019; Bun et al., 2023; Blumen et al., 2021). Multi-morbidity demonstrated a similar association; each one-point increase in the aCCI was linked to 15% higher odds of MCR (OR = 1.15, 95% CI: 1.04 to 1.28, and *p* < 0.05). Chronic cardiometabolic and systemic conditions may contribute to microvascular injury, neuroinflammatory activation, and cognitive–motor impairment (Ding et al., 2025; Jha et al., 2018; Che et al., 2024; Penko et al., 2018), which is consistent with the associations observed in the present analysis.

In addition to these non-modifiable factors, several modifiable factors were identified. Poor sleep quality (PSQI ≥7) was associated with nearly two-fold higher odds of MCR (OR = 2.12, 95% CI: 1.04 to 4.29, and *p* < 0.05). In contrast, higher vitamin D status showed a protective association, with each 1 ng/mL increase in 25(OH)D corresponding to approximately 6% lower odds of MCR (aOR = 0.94, 95% CI: 0.89 to 0.99, and *p* < 0.05). Elevated systemic inflammation, indexed by SII_z, was independently associated with increased MCR risk (aOR = 1.65, 95% CI: 1.22 to 2.24, and *p* < 0.05). These results indicate that sleep regulation, vitamin D status, and inflammatory burden are potentially modifiable factors associated with MCR risk. Importantly, their combined pattern indicates a potential mechanistic link, whereby vitamin D may influence MCR partly through systemic inflammation, thereby supporting the subsequent mediation analysis.

MCR in late life appears to arise from interacting biological and clinical processes, including aging, multimorbidity, sleep disturbance, vitamin D deficiency, and systemic inflammation. These factors may collectively reduce cognitive reserve and disrupt motor–cognitive integration. The mediation observed along the 25(OH)D → SII → MCR pathway further supports the contribution of inflammatory processes to MCR vulnerability. However, the directionality of this pathway cannot be definitively established in a cross-sectional design. Although an alternative pathway (i.e., SII influencing 25(OH)D and subsequently MCR) is theoretically possible, existing evidence more strongly supports a model in which vitamin D modulates inflammatory responses. Accordingly, 25(OH)D was specified as the exposure and SII as the mediator based on biological plausibility and study objectives, and this assumption should be interpreted with caution. Despite these limitations, the findings support risk stratification and highlight potential targets for multi-domain intervention in older adults.

### 4.2 Mechanistic pathway: 25(OH)D → SII → MCR

Serum 25(OH)D is a widely accepted indicator of vitamin D status, and reduced levels of 25(OH)D have been associated with cognitive decline, gait impairment, and increased vulnerability to motor–cognitive dysfunction (Uchitomi et al., 2020). Accumulating evidence indicates that systemic inflammation plays an important role in neurodegenerative and motor–cognitive processes that are relevant to MCR (Lind et al., 2021). Vitamin D regulates inflammatory responses and immune signaling, and its deficiency may promote a pro-inflammatory state (Argano et al., 2025; Bauerle et al., 2021). Such inflammatory activation may adversely affect neural and vascular pathways involved in cognition and gait, thereby providing a biologically plausible link between vitamin D status, systemic inflammation, and MCR. Consistent with this framework, previous studies have reported that inflammatory markers are associated with cognitive impairment and vascular indicators of brain health, further supporting the role of inflammation in motor–cognitive decline (Pan and Ma, 2024). In addition, prior studies have demonstrated associations between low vitamin D levels and MCR-related phenotypes, along with links between heightened inflammatory activity and increased MCR risk (Le Floch et al., 2022). Experimental evidence further indicates that vitamin D modulates inflammatory signaling through the VDR–NF-κB axis (Chen et al., 2013). Beyond this classical pathway, emerging evidence indicates that vitamin D may exert biological effects through alternative metabolic pathways and receptor systems. For example, non-classical vitamin D metabolites generated via CYP11A1-mediated pathways have been reported to possess anti-inflammatory and anti-oxidative properties. Moreover, interactions with other nuclear receptors may contribute to the immunomodulatory and neuroprotective effects of vitamin D. These observations suggest that the biological actions of vitamin D are multifaceted and extend beyond the classical pathways discussed in this study. The SII, which integrates neutrophil, lymphocyte, and platelet counts, has also demonstrated value as a composite indicator of systemic inflammatory burden (Hu et al., 2014).

In this context, we examined whether systemic inflammation mediates the association between 25(OH)D and MCR. Higher 25(OH)D levels were associated with lower SII_z (β = −0.026, 95% CI: −0.042 to −0.010, and *p* < 0.05), and SII_z was independently related to higher odds of MCR (aOR = 1.65, 95% CI: 1.22–2.24, and *p* = 0.001). Mediation analysis demonstrated a significant indirect effect (NIE = −0.043, 95% CI: −0.080 to −0.014) and a proportion mediated of 26.0% (95% CI: 9.9% to 43.2%), with consistent findings across sensitivity analyses. Stratified mediation analysis indicated state dependence; among individuals with low 25(OH)D (≤20 ng/mL), the indirect effect remained significant (NIE = −0.106, 95% CI: −0.194 to −0.027), with a similar proportion mediated (26.8%). In contrast, no significant indirect pathway was observed in participants with higher 25(OH)D. These findings suggest that systemic inflammation amplifies the association between vitamin D deficiency and MCR and that susceptibility to inflammatory influences on motor–cognitive function may be heightened under conditions of low vitamin D status.

Vitamin D deficiency may reduce the threshold for inflammatory activation, while elevated SII_z reflects a pro-inflammatory milieu capable of affecting neural pathways involved in gait regulation and executive control. Together, these results outline a biologically coherent and statistically supported pathway linking the vitamin D status, systemic inflammation, and MCR. They also indicate potential benefits from strategies addressing both vitamin D insufficiency and inflammatory burden in older adults while underscoring the need for longitudinal and interventional research to clarify causal direction.

### 4.3 Limitations and future directions

This study has several limitations. First, the cross-sectional design precludes establishing temporality, and causal relationships cannot be inferred. Reverse causation also cannot be excluded as individuals with poorer health status, reduced mobility, or limited outdoor activity may have lower vitamin D levels due to decreased sun exposure and related lifestyle factors. Second, although blood sampling was performed within 48 h of admission, inpatient treatments—such as vitamin D supplementation or anti-inflammatory medications—may have influenced biomarker levels, and residual confounding cannot be excluded. In particular, factors such as nutritional status, metabolic comorbidities, medication use, and seasonal variation in vitamin D levels were not fully accounted for. Third, MCR was defined using a pragmatic clinical assessment that may introduce misclassification, and single measurements of 25(OH)D and SII may not capture short-term biological variability. In addition, relevant lifestyle and psychosocial factors, including diet, outdoor activity, and depressive symptoms, were unavailable. Furthermore, CRP data were not available in the present study; therefore, comparisons between SII and conventional inflammatory markers could not be performed. Finally, the single-center inpatient sample limits generalizability to community-dwelling populations and other clinical settings. Longitudinal or interventional studies with repeated measurements of 25(OH)D and SII are needed to establish temporal ordering and assess the stability of the observed mediation pathway. Incorporating multimodal evaluation—including neuroimaging, cognitive testing, and objective gait assessment—may help clarify the underlying mechanisms. Stratified analyses based on vitamin D status and inflammatory burden, along with targeted trials of vitamin D repletion with or without anti-inflammatory components, may help identify subgroups most likely to benefit from intervention. In parallel, the development and external validation of parsimonious screening models that integrate 25(OH)D, SII, and readily obtainable clinical features may support translation into risk stratification and early prevention strategies.

## 5 Conclusion

In this single-center inpatient cohort of 312 older adults (January–June 2025), older age, higher aCCI, poor sleep quality, lower 25(OH)D, and higher SII_z were independently associated with MCR. Mediation analysis further indicated that systemic inflammation partially mediates the association between 25(OH)D and MCR, with the effect most pronounced among individuals with 25(OH)D ≤ 20 ng/mL. These findings support a vitamin D–inflammation pathway contributing to motor–cognitive vulnerability and indicate that routinely available indicators may aid in early risk stratification. Future longitudinal and interventional studies are needed to clarify temporal relationships and determine whether vitamin D repletion, alone or combined with anti-inflammatory strategies, can reduce the risk of MCR in high-risk groups.

## Data Availability

The raw data supporting the conclusions of this article will be made available by the authors, without undue reservation.
